# Working memory test battery for young adults: Computerized working memory assessment

**DOI:** 10.1371/journal.pone.0175047

**Published:** 2017-03-31

**Authors:** Liang Ma, Lei Chang, Xiaoying Chen, Renlai Zhou

**Affiliations:** 1 School of Psychology, Beijing Normal University, Beijing, China; 2 Department of Psychology, University of Macau, Macau S.A.R., China; 3 Department of Psychology, School of Social and Behavior Sciences, Nanjing University, Nanjing, China; Universidade Federal do Rio de Janeiro, BRAZIL

## Abstract

This study developed a battery of computerized working memory (WM) tests and a scoring system suitable for young adult users. The tests comprised five classic tasks derived from Baddeley’s model of WM, reflecting each of the five WM functions. We recruited 115 undergraduate and graduate students from various academic fields and constructed a preliminary WM scoring norm for young adults. The scoring norm was used as a basis for developing a computerized assessment system. The results of correlation analysis show that the fluid intelligence of young adults is related to the memory function of WM, but not to the central executive system. The proposed working memory test battery for young adults comprehensively reflects the WM capacity of adults.

## Introduction

### 1.1. State of the art

Working memory (WM) is a cognitive system with limited capacity that enables the temporary storage and manipulation of information. WM is necessary for such complex tasks as comprehension, learning, and reasoning [1, 2], and comprises the following three components: the phonological loop, visuospatial sketch pad, and central executive system. The phonological loop is a temporary storage system in which acoustic or speech-based information can be held as memory traces that spontaneously fade. The visuospatial sketch pad temporarily stores visual and spatial information. The central executive is responsible for attentional control and information processing pertaining to WM [3]. The phonological loop and visuospatial sketch pad comprise the information storage system of WM, and the central executive serves as the information processing system of WM. According to the operation of WM information in the storage system, the central executive is divided into the following three distinct yet strongly interactive functions: (1) controlling shifting between tasks or mental sets (i.e., shifting function); (2) controlling the inhibition of proponent responses (i.e., inhibition function); and (3) controlling the updating and monitoring of WM representations (i.e., updating function) [4]. WM correlates highly with fluid intelligence [5–7] and reasoning ability [8–10]. Jaeggi trained the WM capacity of adults through a dual n-back task [11]. Zhao trained 9–11-year-old children through running WM tasks [12]. Rudebeck trained young adults using a spatial WM training task [13]. Wang trained children with a running WM task [14]. All of these studies have shown increases in fluid intelligence, thereby demonstrating the close relationship between WM development and fluid intelligence.

WM is also closely related to decreases in human cognitive abilities. Therefore, if we develop a WM assessment for adults, researchers can estimate the severity of disease from a cognitive perspective. In neuropathological research on Alzheimer’s disease, the cognitive decline associated with dementia has provided subtle evidence of a decline in WM capacity [15]; the higher WM capacity a person has, the slower the decline in his or her cognitive ability during the early stage of the disease [16]. In addition, other cognitive diseases such as attention deficit hyperactivity disorder [17], schizophrenia [18], time estimation disorder [19], reading disorders [20], and emotional regulation disorder [21, 22] correlate highly with WM capacity. Because of the importance of WM, research on WM measurement has attracted considerable attention in recent years. Gathercole et al. developed a set of WM tests for children aged 6–7 years based on Baddeley’s three-component model. However, of the aforementioned three WM components, the test was effective only for measuring the phonological loop [23]. In recent years, an increasing number of WM test batteries have been produced. Kenny and Hicks [24] developed a WM capacity test set called Online Working Memory Lab (OWL), which consisted of the following four complex span tasks in its first version: operation span, reading span, symmetry span, and running span [25]. In its most recent version [24], patterns were used as task material instead of letters, which were more difficult to write in unsupervised settings. Because the changing of memory material prevented cheating, the test results were as reliable as laboratory results.

Van [26] developed a set of visual WM test procedures for children aged 6–12 years that is similar to a visual version of a running memory task called “Lion Game.” In each trial, eight lions of different colors were displayed consecutively at different locations in a 4 x 4 matrix. The children were asked to remember the last location where a lion of a certain color had appeared and use the mouse to click on that location after the sequence had ended. The task consisted of five levels, and WM load was scored based on the number of colors (i.e., locations) that the children had to remember and update. The most notable features of this task were the self-reliant administration design and web browser—based format. However, the selection element of the task was too simple, and thus the testing effect was limited in the following manners: First, the task was not a test of pure visual span; with the mission ongoing, each child had to continually update his or her memory to recall the preceding locations, which served as indicators of the updating function in the central executive function. Second, a mouse was required, rendering it difficult to accurately record reaction times, which are a crucial indicator for evaluating WM.

Oswald [27] developed a shortened computerized measure of WM capacity by representatively sampling items from operation span, reading span, and symmetry span tasks. Although these tasks were similar to those employed in other studies, notably, the test time was shortened.

In summary, the main features of the WM test battery are as follows: (1) Shorter time; implementation of WM measures is generally time-consuming, and examinees and researchers are often required to manage the tension between limited testing time and the need to reliably measure numerous constructs. A short and effective measure of WM capacity could be a major practical benefit in the future [27]. Previous studies have also proven that shortened test tasks can effectively test WM ability [28]. (2) A computerized or web-based format, thereby facilitating the collection of high quantities of data. The most significant research obstacle is that the quality of collected data is unsatisfactory. Without the supervision of research assistants, online task participants may cheat.

We proposed a test with a simple and clear paradigm to test all WM components within a short period of approximately 30 minutes. Because our test was based on a local computer and conducted entirely through keyboard operation, no network latency or response time bias could be caused by operational problems. This test program can not only record the accuracy of each task, but also accurately record the reaction time of each trial to evaluate WM capacity more accurately. Several general limitations of WM tests are defined in the following section.

### 1.2. Limits of the current literature

The limitations of extant research on WM tests are described as follows.

1. The literature lacks complete sets of comprehensive tests. Tests of WM components are either relatively homogeneous or involve test tasks that do not clearly reflect the functions of specific components. Langer designed the “tower of fame” task and argued that in addition to the relational integration component of WM [29], successful completion of this task may also be related to the storage function. The only task used in the WM test by Siegel was the auditory span test, which does not reflect overall WM ability [30]. León-Domínguez recently used an improved version of the n-back task to measure the central executive function [31], but this approach does not measure the inhibitory function or switch function. The more widely recognized WM test sets currently in use are the Automated Working Memory Assessment (AWMA) [32]and Working Memory Index in the Wechsler Intelligence Scale for Children, Fourth Edition (WISC-IV) [33]. Moreover, some scales pertaining to WM such as the Working Memory Rating Scale [34], Behavior Rating Inventory of Executive Function [35], and Conners’ Teacher Rating Scale [36] are not cognitive tests, but are rather questionnaires designed mainly for students. According to teachers’ responses to questionnaires, researchers can determine whether students’ WMs are defective. However, the results of this approach are influenced by the teachers’ subjective feelings, and thus do not reflect the students’ WM potential [37]. The establishment of the tests in the present study was based on the Baddeley’s three-component model of WM [1, 2], which has been thoroughly tested and is widely accepted as a reliable model among researchers. In Baddeley’s model, the phonological loop, visuospatial sketch pad, and central executive (including inhibition, switching, and updating functions) are measured. A participant’s final score, which is derived from five subtest scores, provides a clear and comprehensive indication of his or her overall WM capacity.
2. Evaluation criteria are ambiguous. Most WM tests adhere to the original score level, thereby creating the problem that using the task accuracy as a direct indicator impairs comparability between tests. In the present study, all of the test scores were standardized to enable comparisons between various studies. Each participant’s test score could be converted into a derived score after completion of the measurement to illustrate the degree of his or her capability.
3. A lack of large-scale group tests and score norms render further comparison of the performance of WM tests impossible. With the exception of the AWMA and WISC-IV, which contain scoring norms, a regular norm for WM tests has not been established. One aim of the present study was to establish an appropriate test norm for young adults with a view to constructing an embryonic national model for future reference.
4. Few computerized WM tests have been developed; consequently, tasks can be applied for individuals but not for groups, yielding low test efficiency. Berg used a visual matrix task, the Corsi block-tapping test, semantic classification, and an auditory digit sorting task to examine the relationship between WM and mathematical ability [38]. The tasks were difficult to program and operate on a computer screen, which influenced the test results. Moreover, (verbal) digit sort, (verbal) digit-span backward, the WISC-IV Working Memory Index, AWMA, and other practical tasks are prone to similar problems. All the experimental tasks in the present study were computerized to ensure that the experimenter effect would be avoided and reaction times would be recorded accurately.

### 1.3. Study objective

The aim of the present study was to establish a set of effective WM capacity evaluation tools and scoring norms suitable for adults.

The aforementioned studies suggest that cognitive ability, health status, and daily life are closely related to WM ability. Moreover, a survey of WM ability seems necessary. Regarding cognitive ability, a battery of WM tests could provide a more comprehensive scale for measuring information processing ability, which would be a viable predictive intelligence index for personnel assessment and selection. Regarding disease screening, a decline in WM is an early warning sign of mental disorders such as schizophrenia [39], hyperactivity, senile dementia [14, 15, 40], and insomnia [41]. Furthermore, WM capacity test results can be used to guide further cognitive intervention for individuals with certain weak WM components, thereby ensuring that such individuals receive well-directed training on those components. Theoretically, studying the composition and functions of WM as well as the development or decline of cognitive ability is crucial in forming a set of indicators to describe the various abilities of WM.

The test presented in this study can be applied not only in cognitive ability assessments—which can be used in school entrance examinations and personnel selection and assessment procedures—but also for the rapid diagnosis of psychological disorders. The scores for the proposed set of WM tests were standardized to avoid confusion due to different test tasks and test indices, thereby rendering the scores comparable for humans.

## Methods

### 2.1. Participants

In this study, 115 college students (mean age: 22 ± 3.47 years; 62 females) were enrolled in Beijing and Nanjing. They all reported strong unassisted or corrected vision and standard color perception, and reported no history of psychiatric or neurological diseases, neuropsychological problems, or substance abuse. Each subject signed an informed consent form and received a reward after completing all the tests. The study was approved by the Beijing Normal University Ethics Committee.

### 2.2. Measurements

#### 2.2.1. WM measurements

The test programs were conducted using computers; therefore, the duration of each trial and interval between consecutive trials were designed precisely according to a program statement. The rules of each test were simple, methods of data collection and analysis were convenient, and the test was suitable for group testing. The entire set of tests comprised the following five subtests that correspond to specific WM components: (1) The visuospatial sketchpad function was tested using a visual delayed match-to-sample task, (2) the phonological loop was tested using a letter-delayed match-to-sample task, (3) the switching function was measured through a number-switching determination task, (4) the updating function was measured using a running WM task, and (5) the inhibition function was measured using the numerical Stroop task. These tasks were selected because (1) the preparation, rules, and completion of the tasks were simple, rendering them easy for experimenters and participants to understand; (2) the tasks were considered “pure” for measuring the corresponding components of WM [42], had been used extensively, and had high content validity; and (3) the tasks were appropriate for computer software, and thus were conducive for conducting various assessments and collecting data.

**Fig 1:**
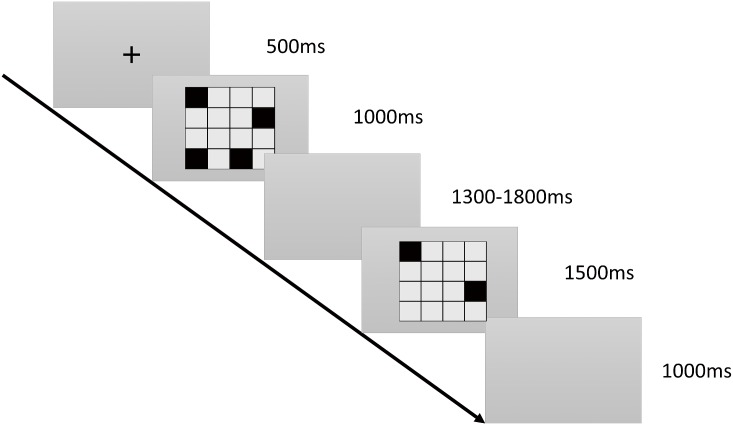
Visuospatial sketchpad subtest. Time course of the stimulus presentation in the visual delayed match-to-sample task. A black “+” symbol is displayed at the center of the screen for 500 ms as a reminder to the participants to pay attention. In the 4 × 4 grid, 4–9 black squares are displayed in a random order for 1,000 ms each. The participants must remember the locations of the black squares. Subsequently, the black squares disappear, and the screen remains blank for approximately 1,500 ms before the grid appears again with only two black squares. Participants must determine whether these two black squares are located in the same positions as any of the previously displayed 4–9 black squares.

**2.2.1.1. Visuospatial sketchpad subtest:** In this task, a 4 × 4 grid is displayed at the center of the screen. Each participant must memorize the locations of 4–9 black squares. Subsequently, the screen is cleared and another two black squares appear on the screen. The participant must determine whether the latter two squares are located in the same position as any of the previously displayed black squares. The more squares there are to be memorized, the more difficult the task is. Participants who complete the task in shorter times and with higher accuracy are considered to have stronger visuospatial sketchpad functions, and thus higher visual WM capacities. The details of the subtest are showed in Fig 1.

**Fig 2:**
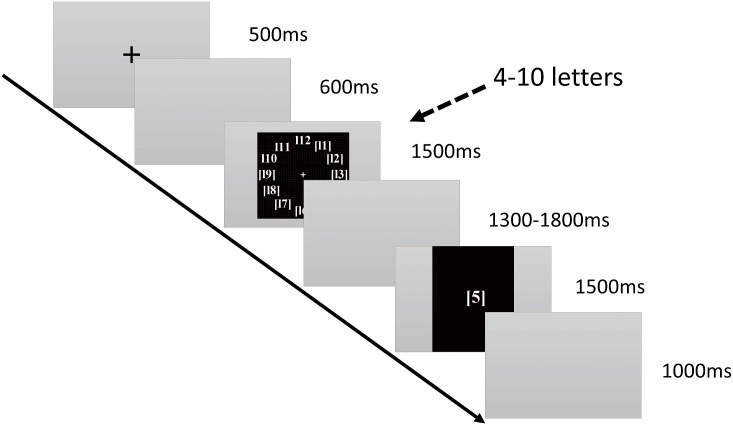
Phonological loop subtest. Time course of the stimulus presentation in the letter delayed match-to-sample task. A black “+” symbol is displayed at the center of the screen for 500 ms as a reminder to the participants to pay attention. Subsequently, a blank screen is displayed for approximately 600 ms before a letter loop appears. In a clock-like loop, 4–12 upper case letters are displayed in a random order for 1,500 ms each. After the letter loop disappears, the screen remains blank for 1,300–1,800 ms before a lowercase letter appears at the center of the screen for a further 1,500 ms. Participants must determine whether this letter was among the letters in the clock-like loop (irrespective of case). Subsequently, another blank screen is displayed for 1,000 ms, after which the task ends.

**2.2.1.2. Phonological loop subtest:** In this task, a circle formed by 4–12 uppercase letters is presented at the center of the screen. Each participant must memorize the uppercase letters. Subsequently, another lowercase letter is displayed in the screen. The participant must determine whether the same letter was among the previously displayed uppercase letters (irrespective of case). The more uppercase letters there are to be memorized, the more difficult the task is. Participants who complete the task in shorter times and with higher accuracy are considered to have stronger phonological loop function, and thus, higher verbal WM capacities. The details of the subtest are showed in Fig 2.

**Fig 3:**
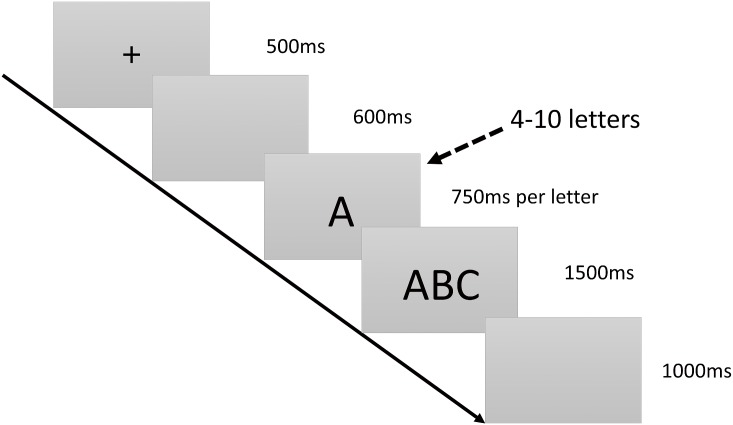
Updating function subtest. Time course of the stimulus presentation in the running WM task. A black “+” symbol is displayed at the center of the screen for 500 ms as a reminder to the participants to pay attention. Subsequently, a blank screen is displayed for approximately 600 ms before a letter series appears. The letter series contains 4–12 uppercase letters that appear sequentially, with each letter being displayed for 750 ms. After the letter series disappears, three of the letters reappear at the center of the screen for approximately 1500 ms. Participants must determine whether these three letters were the last three letters of the previously displayed series. Subsequently, another blank screen is displayed for 1,000 ms, after which the task ends.

**2.2.1.3. Central executive system: Updating function subtest:** In the letter delayed version of the running WM task, a series of letters is sequentially displayed at the center of the screen. Each series contains a different number of letters. Participants must memorize the last three letters that appear. Subsequently, another three letters are displayed on the screen simultaneously. Participants must determine whether the three letters match the three letters they have memorized. The more letters in a series, the more difficult the task is. Participants who complete this task in shorter times and with higher accuracy are considered to have stronger updating functions. The details of the subtest are showed in Fig 3.

**Fig 4:**
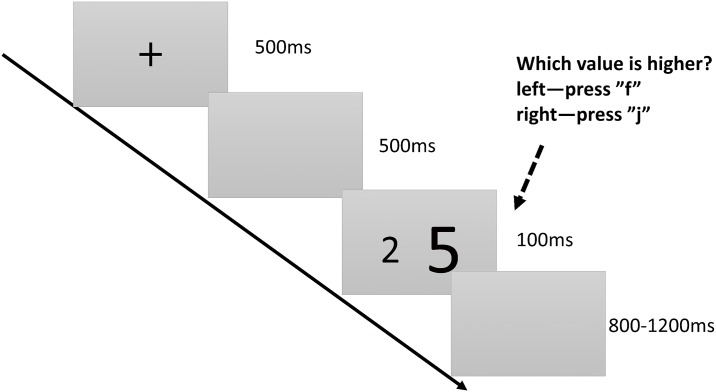
Inhibition function subtest. Time course of the stimulus presentation in the number-size Stroop task. A black “+” symbol is displayed at the center of the screen for 500 ms as a reminder to the participants to pay attention. Subsequently, a blank screen is displayed for approximately 500 ms before two numbers appear for 100 ms each. Participants must determine which number has the higher value while ignoring the digit sizes. After another blank screen is displayed for 800–1,200 ms, the task ends.

**2.2.1.4. Central executive system: Inhibition function subtest:** The inhibition function can be measured through a number-size Stroop task [4], where two differently sized Arabic numerals are displayed on the screen simultaneously. Each participant must determine which number has the higher value while ignoring their sizes. According to the visual reaction characteristics of individuals, when determining two-digit number values, one should inhibit prepotent responses. In this task, the congruent condition is that the value of the larger sized digit is greater. The incongruent condition is that the value of the smaller sized digit is greater, forcing participants to restrain their prepotent responses to make accurate judgments. The neutral condition is that both digits are congruent in size but incongruent in value. A participant’s reaction time difference between the incongruent and neutral conditions was considered as an index of the inhibition function. Participants who complete this task in shorter times and with higher accuracy are considered to have stronger inhibition functions. The details of the subtest are showed in Fig 4.

**Fig 5:**
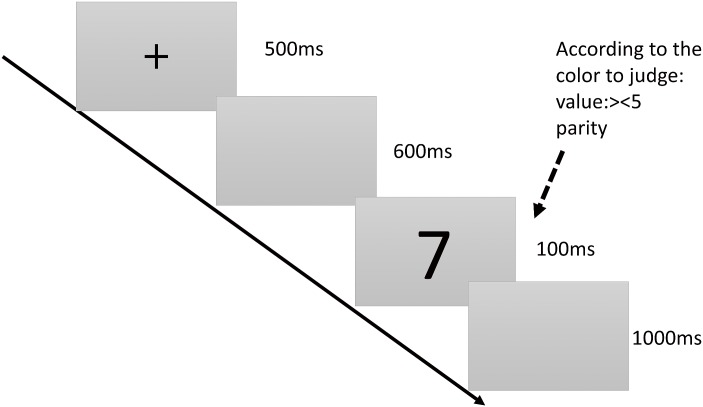
Switching function subtest. Time course of the stimulus presentation in the number-switching task. A black “+” is displayed at the center of the screen for 500 ms as a reminder to the participants to pay attention. Subsequently, a blank screen is displayed for approximately 600 ms before a number appears at the center of the screen. Each number appears for 100 ms. The participants must determine whether the values of the red numbers are higher than 5 and whether the blue numbers are odd or even. Subsequently, another blank screen is displayed for 1,000 ms, after which the task ends.

**2.2.1.5. Central executive system: Switching function subtest:** This subtest involves two single tasks to determine digit value and parity, and one dual task to determine the values of red digits and parities of blue digits. When the mean reaction time of the dual task differs from that of the single task, the difference is regarded as an index of switching ability. Participants who complete this task in shorter times and with higher accuracy are considered to have stronger switching functions. The details of the subtest are showed in Fig 5.

#### 2.2.2. Fluid intelligence measurement

All participants completed a computerized version of Raven’s Advanced Progressive Matrices (APM) test. The test scores were recorded as reflections of the participants’ fluid intelligence.

### 2.3. Processing

Because the test set in the present study was based on a computer program, the process was strictly standardized, thereby almost eliminating operational errors, and enabling the reaction times to be recorded more precisely. All of the tests were conducted in standard behavioral laboratories or computer classrooms. The computer screen display resolution was set to 1366 × 768 and the brightness was adjusted to 100%. To avoid the order effect influencing the results, we randomized the order of the five subtests in the WM test battery. All participants undertook the APM test after completing the computerized WM test battery.

## Results

### 3.1. Descriptive statistics

SPSS 22.0 was used to analyze the data. Table 1 provides descriptive statistics for the whole sample.

**Table 1:** Descriptive statistics among observed variables for the whole sample (n = 115).

Task	Group	M ± SD	Skewness	Kurtosis
Visual delayed match-to-sample task (visuospatial sketchpad)	ACC	0.76 ± 0.11	-0.73534	0.669303
	RT	1139.54 ± 148.37	0.179744	-0.25576
Letter-delayed match-to-sample task (phonological loop)	ACC	0.83 ± 0.06	-0.55522	0.070389
	RT	947.61 ± 155.38	0.562865	0.614193
Running WM task (updating function)	ACC	0.93 ± 0.06	-1.78034	4.394886
	RT	78.22 ± 29.17	0.444423	0.346707
Number-size Stroop task (inhibition function)	ACC	0.82 ± 0.14	-2.62499	9.254913
	RT	1035.16 ± 143.64	-0.18702	-0.11504
Number-switching determination task (switching function)	ACC	0.91 ± 0.06	-0.6326	-0.47601
	RT	361.94 ± 142.46	2.69902	16.36699

*Note*: ACC = accuracy, RT = reaction time.

Table 2 provides correlations among observed variables for the whole sample, showing high correlation among the subtests of the battery assessment.

**Table 2:** Correlations among observed variables for the whole sample (n = 115).

	1	2	3	4	5	6	7	8	9	10
1 VACC	1									
2 VRT	.186*	1								
3 PACC	.432**	.070	1							
4 PRT	-.004	.587**	-.070	1						
5 IACC	.009	.314**	-.046	.363**	1					
6 IRT	-.115	.003	-.089	.008	.030	1				
7 UACC	.381**	.038	.207*	-.127	.108	-.097	1			
8 URT	-.087	.546**	-.188*	.566**	.236*	.132	-.174	1		
9 SACC	.192*	.218*	.078	.122	.404**	.072	.168	.082	1	
10 SRT	-.065	.066	-.218*	.048	.134	-.072	.009	.165	.251**	1

Note: n = 115; VACC = accuracy of visual delayed match-to-sample task; VRT = reaction time of visual delayed match-to-sample task; PACC = accuracy of letter-delayed match-to-sample task; PRT = reaction time of letter-delayed match-to-sample task; IACC = accuracy of Number-size Stroop task; IRT = reaction time of Number-size Stroop task; UACC = accuracy of running WM task; URT = reaction time of running WM task; SACC = accuracy of number-switching determination task; SRT = reaction time of number-switching determination task;

*P < 0.05,

**P < 0.01.

### 3.2. Discrimination analysis

The 27% of the participants with highest scores and 27% with the lowest scores were selected as high-score and low-score groups (n = 31 in each). An independent sample *t* test was conducted between the two groups, revealing significant differences between the tested groups, thereby establishing discrimination in the computerized test battery. The results are shown in Tables 3 and 4.

**Table 3:** Accuracy differences between high- and low-score groups (%).

Task	Group	M ± SD	*t*
Visual delayed match-to-sample task (visuospatial sketchpad)	High-score	88.74 ± 2.79	-18.238**
	Low-score	62.31 ± 7.57	
Letter delayed match-to-sample task (phonological loop)	High-score	89.63 ± 2.27	-19.084**
	Low-score	75.20 ± 3.54	
Running WM task (updating function)	High-score	93.98 ± 2.46	-8.807**
	Low-score	65.62 ± 17.76	
Number-size Stroop task (inhibition function)	High-score	98.74 ± 0.92	-12.055**
	Low-score	85.30 ± 6.52	
Number-switching determination task (switching function)	High-score	97.26 ± 1.23	-20.388**
	Low-score	82.38 ± 2.5	

*Note*: *P < 0.05,

**P < 0.01.

**Table 4:** Reaction time differences between high- and low-score groups (ms).

Task	Group	M ± SD	*t*
Visual delayed match-to-sample task (visuospatial sketchpad)	High-score	961 ± 66	20.323**
	Low-score	1330 ± 75	
Letter delayed match-to-sample task (phonological loop)	High-score	770 ± 62	17.259**
	Low-score	1144 ± 104	
Running WM task (updating function)	High-score	861 ± 83	19.108**
	Low-score	1211 ± 59	
Number-size Stroop task (inhibition function)	High-score	44 ± 11	17.943**
	Low-score	115 ± 19	
Number-switching determination task (switching function)	High-score	226 ± 49	9.988**
	Low-score	527 ± 160	

*Note*: *P < 0.05,

**P < 0.01.

### 3.3. Reliability analysis

Across the whole sample, the split-half reliability for accuracy was 0.673, and that for reaction time was 0.614; both were calculated using the Spearman—Brown prediction formula. The Cronbach's alpha of accuracy and reaction time across the whole sample were 0.529 and 0.625, respectively, thereby establishing the internal reliability of the scale.

### 3.4. Validity analysis

#### 3.4.1. Construct validity

Exploratory factor analysis (EFA) was conducted with varimax rotation and the principal component extraction method, the results of which are shown in Table 5. Only components with eigenvalues of >1 were selected. The scores of the 10 test indicators (five accuracies and five reaction times) revealed the following four factors: storage system reaction time (Factor 1), storage system accuracy (Factor 2), central executive system accuracy (Factor 3), and central executive system reaction time (Factor 4), which cumulatively explained 68% of the variance. Each index loading in its respective dimension ranged from 0.5 to 1.

**Table 5:** EFA results for the participants’ accuracy and reaction times in the five subtests.

	Factor 1	Factor 2	Factor 3	Factor 4
Phonological loop RT (PRT)	0.86			
Visuospatial sketchpad RT (VRT)	0.83			
Updating RT (URT)	0.80			
Visuospatial sketchpad ACC (VACC)		0.8		
Phonological loop ACC (PACC)		0.77		
Updating ACC (UACC)		0.58		
Switching ACC (SACC)			0.79	
Inhibition ACC (IACC)			0.62	
Switching RT (SRT)			-0.64	-.399
Inhibition RT (IRT)				0.91

*Note*: ACC = accuracy, RT = reaction time.

**Fig 6:**
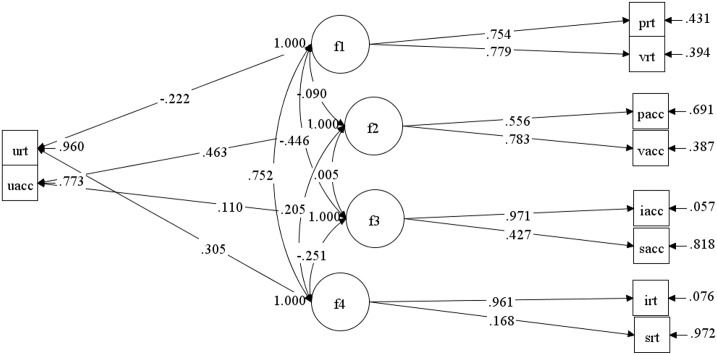
Path model and standardized coefficients of confirmatory factor analysis.

Confirmatory factor analysis (CFA) was employed to evaluate the relative fit of the four-factor WM model by using Mplus 7.0. Table 6 presents the indices of the fitting results between the test sets of the four-factor model and Baddeley’s three-component model of WM. The path model for the four-factor model is shown in Fig 6.

**Table 6:** Fitness of the CFA model.

	χ^2^	*df*	*P*	RMSEA	CFI	TLI
Model	38.545	27	0.0696	0.061	0.941	0.902

*Note*: RMSEA = root mean square error of approximation, CFI = comparative fit index, TLI = Tucker—Lewis Index.

A commonly used index for the goodness of fit of a model is the χ^2^ value, which compares the degree to which the predicted covariance in a model differs from the observed covariance. Adequate fitness is usually determined by low, nonsignificant χ^2^ values. Because this index is sensitive to the sample size, very large samples (as in the present study) cause even the best-fitting models to frequently yield significant χ^2^ values [43]. Therefore, additional global fit indices that are more sensitive to the model specification were used to indicate the fitness of the model. Fit indices such as the comparative fit index (CFI) [44], Bollen’s incremental fit index (IFI) [45], and the root mean square error of approximation (RMSEA) provide further measures of fit by comparing the hypothesized model against a null model in which the relations between the latent variables are not specified and are consequently set at 0. Fit indices with values equal to or higher than 0.90 demonstrate a good fit. In addition, an RMSEA of <0.09 demonstrates a good fit. Table 6 shows that the fit indices met the basic criteria requirements, indicating that the test in the present study exhibited acceptable construct validity.

#### 3.4.2. Convergent validity

Regarding the use of the APM test scores as criteria, Table 7 shows high correlations between the storage function test scores and APM scores, indicating that the assessment tools’ calibration validity met the requirements of the basic criteria.

**Table 7:** Pearson’s correlation coefficients for WM measures and Raven’s APM accuracy.

Task	*r*
Visual delayed match-to-sample task (ACC)	0.424**
Visual delayed match-to-sample task (RT)	-0.063
Letter delayed match-to-sample task (ACC)	0.255**
Letter delayed match-to-sample task (RT)	-0.178**
Running WM task (ACC)	0.092
Running WM task (RT)	-0.039
Number-size Stroop task (ACC)	-0.029
Number-size Stroop task (RT)	-0.167
Number-switching determination task (ACC)	0.095
Number-switching determination task (RT)	-0.014

*Note*: *P < 0.05,

**P < 0.01,

ACC = accuracy, RT = reaction time.

### 3.5. Common method variance

In this study, we used the Harman one-factor test method to test for common method variance. The results are shown in Table 8.

**Table 8:** Total variance explained.

Component	Initial eigenvalues	Total % of variance	Cumulative %
**1**	**2.512**	**25.125**	**25.125**
**2**	**1.905**	**19.046**	**44.171**
**3**	**1.29**	**12.902**	**57.073**
**4**	**1.041**	**10.406**	**67.48**
5	0.802	8.021	75.501
6	0.738	7.385	82.885
7	0.508	5.077	87.962
8	0.463	4.632	92.595
9	0.38	3.801	96.396
10	0.36	3.604	100

Table 8 shows that Factor 1 accounted for approximately 25%, which is less than half of the 67% of the cumulative variance accounted for by Factors 1–4. The CFA results verifying the one-factor model are shown in Table 9.

**Table 9:** Comparison of fitness indices between the one-factor model and original model.

	χ^2^	*df*	*P*	RMSEA	CFI	TLI
Original	38.545	27	0.0696	0.061	0.941	0.902
One-factor	186.623	35	<0.001	0.194	0.228	0.007

*Note*: $\Delta \chi^{\text{2}}=148.078>\Delta \chi_{\alpha =0.05}^{2}\left(8\right)=15.51$.

The difference between the two models is significant, with the original model providing a significantly closer fit to the data than the one-factor model. Therefore, common method bias should not affect the interpretation of the results, indicating that the tests have adequate validity and the data is relatively suitable for analysis.

### 3.6. Norm establishment and fractional conversion

The accuracy index was the integral index of the visuospatial sketchpad, phonological loop, and updating function tests. However, in the actual tests, the reaction times for these three tasks were recorded as a secondary index for reference. The inhibition and switching function indices, which were calculated through the subtraction rule, were the absolute values of the reaction time difference during the specific cognitive process. These scores were standardized using their opposite numbers, resulting in proportional changes in the models of reaction time and accuracy. In other words, the greater the fractional conversion value, the stronger the capacity of the project is. However, for these two tasks, the accuracy in the actual tests was recorded as a supplementary reference. In the WM tests, each subtest score was converted into a *z*-score to obtain the participants’ overall WM scores. In addition, we determined the corresponding percentile ranks of the overall scores in the established scoring norm. However, participants with identical total scores may have had different subtest scores, indicating individual differences that highlight the participants’ strengths and weaknesses in WM.

## Discussion

In each test, significant differences were observed between the accuracy and reaction times of the high- and low-score groups, indicating that the test results reveal differences in reaction quality and speed with high discriminatory power.

Because accuracy and reaction time are two distinct index systems with relatively low internal consistency, the Cronbach’s α values for the participants’ accuracy and reaction times in the five subtests were analyzed. The results were within the acceptable range of 0.673 and 0.614, possibly because of the excessively centralized controlling of the tests’ difficulty levels. As shown in Table 1, the score distribution for most tasks is relatively narrow, and the accuracy of the high scores for the inhibition and switching function tasks exhibited a weak ceiling effect.

### 4.1. Questions regarding the purity of the updating function task

In accordance with the EFA results, the indices were classified into the following four factors: storage function accuracy (Factor 1), storage function reaction time (Factor 2), central executive function accuracy (Factor 3), and central executive function reaction time (Factor 4). However, the switching function task reaction time being classified as Factor 3 might be a result of the EFA’s emphasis on the data. According to the factor loading matrix, a loading of approximately 0.4 was found for the switching value in Factor 4, indicating that double loading occurred when EFA was conducted for the reaction time switching function index. This may have been due to the greater dependency of the data or the assumption of the WM component model itself, which requires improvement. In addition, the reliability of the EFA results should be discussed in combination with the CFA results. Nevertheless, the accuracy and reaction time indices of the inhibition function tasks were divided into the appropriate storage systems, possibly because of the data model of the updating function tasks being similar to those of the visuospatial sketchpad and phonological loop tasks. Although Morris and Jones demonstrated that the performance of a running WM task is determined by the central executive function rather than the storage function, the updating function is more closely related to the storage function than the other two central executive functions are[46]. Morris and Jones argued that the updating task is essentially an updating operation involving the assignment of ordinal tags that are stored in the phonological loop of the storage function and must be constantly updated and repeated. By contrast, in the Stroop tasks, only the inhibition of the familiar operation reaction was conducted, of which the targets were mostly reliant on long-term memory. For example, the values and sizes of the numbers in the number-size task were derived from the participants’ existing cognition of numbers. Furthermore, the operation targets of the switching task were not stored in the WM storage function. Therefore, the overlap of the updating task necessary for the treatment of the message in the storage functions may actually be unavoidable, which is in agreement with the findings of Ecker and Lewandowsky [47]. The updating function is divided into the following three components: retrieval, transformation, and substitution. Retrieval refers to the retrieval process of updating existing messages, transformation refers to transforming an old retrieved message into a new message, and substitution refers to replacing an old message with a new one. The target of the retrieval process is the message stored in the storage function. Hence, the interpretation of the double loading in the updating function task results is reasonable.

CFA was conducted by combining our results with those of the WM tests and EFA obtained by Baddeley[3] and Miyake[4]. The path model is shown in Fig 6, where f1, f2, f3, and f4 denote Factors 1, 2, 3, and 4, respectively. Based on the EFA results, the double loading between the accuracy and reaction time factors in the central executive function was formed when the switching reaction time was formed, yielding Model M2 (Table 10). Although M2 achieved a good fit, approximating the original model (Model M; $\Delta \chi^{2}=1.94<\Delta \chi_{\alpha =0.05}^{2}\left(1\right)=3.84$) by allocating the reaction time index to the accuracy index is meaningless. Hence, under the same fitting conditions, Model M should be used. In addition, Model M1 was obtained by limiting the loading for the updating function to the central executive function based on Baddeley’s three-component model of WM. Table 10 indicates that Model M1 did not achieve a good fit and was inferior to Model M ($\Delta \chi^{2}=18.135>\Delta \chi_{\alpha =0.05}^{2}\left(2\right)=5.99$), as demonstrated by the EFA results.

**Table 10:** Fit conditions of the various models.

	χ^2^	*df*	*P*	RMSEA	CFI	TLI
M (original model)	38.545	27	0.0696	0.061	0.941	0.902
M1	56.68	29	0.002	0.091	0.859	0.781
M2	36.605	26	0.08	0.06	0.946	0.907

Overall, the test results of this study accord with those of the expected model, with the well-fitting CFA model and adequate construct validity meeting surveying requirements.

### 4.2. Relationship between WM and fluid intelligence in adults

Raven’s APM test was conducted to measure the fluid intelligence of all the participants. Fluid intelligence was used to verify the criterion validity of the WM battery tests. The results show that only the accuracy of the visuospatial sketchpad function and phonological loop function in the storage function were related to the fluid intelligence test, and that no indices in the central executive function correlated significantly with fluid intelligence. According to numerous related studies with child participants, the development of WM in children is the direct basis of the development of their fluid intelligence. Such a close relationship is likely due to the central executive function[12, 48–50]. Nevertheless, the results of the present study differ considerably from those of previous studies, likely because the present study enrolled adult participants. In a study on calculation strategy tests for children, teenagers, and young adults that involved tracking functional magnetic resonance imaging, Qin showed that older people tend to rely more on the information storage area of the brain (hippocampus) when solving problems, and less on the information processing area of the brain (prefrontal lobe) [51]. After reaching adulthood, an individual’s cognitive strategies and problem-solving mechanisms no longer focus on the quality of information processing, but on the invocation of information stored in a more mature brain. Although the central executive function in adults might not be outstanding, they possess sufficient storage capacity or memory capacity to solve problems after simply processing information. Based on the results obtained from participants who completed the testing tasks in this study, fluid intelligence performance is related to storage function performance. Therefore, the storage function of WM in adults is more closely related to fluid intelligence, possibly because of adults’ greater dependence on cognitive strategies for problem-solving. Similar findings were reported by Swanson in a study involving children with and without dyslexia selected for cognitive strategy training [52]. The results showed that although the participants’ WM capacities improved, their cognitive abilities after training remained limited by their WM capacities. The aforementioned studies have proven that the more advanced an individual’s problem-solving strategies, the greater the role of the storage function of WM in problem-solving and reasoning is.

The reason that no reaction time indicators were related to the APM test scores was that the fluid intelligence test index measured accuracy. According to the previous discussion, the extent of the variation exhibited by the accuracy and reaction time indices precluded their being related.

The test in the present study had the following deficiencies, and could be improved as follows: The number of participants could be increased to expand the national norm, and the duration of the test was too long, causing the participants to become bored and tired. Therefore, the battery tests should be divided into two or three sections with a break between each section to prevent the participants’ mental states from influencing the results.

## Conclusion

Following Baddeley’s three-component model of WM, the present study employed a visual delayed match-to-sample task, letter-delayed match-to-sample task, running WM task, number-size Stroop task, and number-switching determination task to develop a computerized WM test battery for adults. The test battery can measure the following five WM components reflecting various aspects of WM: the visuospatial sketchpad, phonological loop, and central executive function (i.e., inhibition function, switching function, and updating function). The tests had moderate difficulty levels, and the results show that the tests had high discriminatory power, reliability, and validity. The test battery was conducted using computers to ensure that the test process was more standardized and enabling reaction times to be recorded more precisely, thereby guaranteeing the feasibility of large-scale or web-based tests in the future. A standard scoring norm was established to ensure comparability among the test results. The results revealed disadvantages of Baddeley’s WM model, thereby laying a foundation for further research on WM capacity development.

## Supporting information

S1 File All the data. (RAR)
